# Human brain microglia responses to amyloid beta and tau pathology in high pathology controls and across disease stages

**DOI:** 10.1002/alz.088080

**Published:** 2025-01-03

**Authors:** Mikhail Melnik, Vivianne Mitri, Swetha Atluri, Nina Knitowski, Achyutha Kodavatikanti, Tina Bilousova, Jessica E Rexach, Karen H Gylys

**Affiliations:** ^1^ University of California, Los Angeles, Los Angeles, CA USA; ^2^ University of California, San Francisco (UCSF), San Francisco, CA USA; ^3^ Lawrence Chen Program in Neurogenetics, Department of Neurology, David Geffen School of Medicine, University of California, Los Angeles, CA USA; ^4^ Program in Neurogenetics, Department of Neurology, David Geffen School of Medicine, University of California Los Angeles, Los Angeles, CA USA

## Abstract

**Background:**

Microglia responses to Aβ and tau pathology and the dysregulation of the microglial role in synaptic function may determine the onset and course of Alzheimer’s disease (AD). While significant work has been performed in mouse models, we still lack a complete understanding of physiological and pathological microglial states and functions in human AD brain.

**Method:**

For immunoblotting of brain homogenates against multiple microglial markers, and flow cytometry (FC) analysis of synaptosomal fractions (SNAP25/CD47/Aβ(10G4)/phospho‐tau(AT8)), 49 cryopreserved human parietal cortex samples were categorized into four groups: low pathology control (LPC), high Aβ control (HAC), high pathology control (HPC), and AD. Selected microglia markers were assessed in the snRNAseq dataset (Rexach et al., 2023, bioRxiv) and validated by immunohistochemistry (IHC) analysis.

**Result:**

In the LPC group, only 22.5% of Aβ‐positive (+)/p‐tau‐negative (‐) synaptosomes expressed CD47 on their surface, with a significant progressive increase in the percentage (%) of CD47+ events in the Aβ+/p‐tau‐ group across disease stages, reaching 53.4% in the AD group. Surprisingly, Aβ+/tau+ synaptosomes expressed the highest CD47 levels, suggesting protection from elimination. Higher levels of P2ry12 and CD206 were found in the LPC group compared to all other groups in brain homogenates. Interestingly, the levels of P2ry12, CD206, and Axl1 were significantly higher, and the level of Clec7a was significantly lower in the HPC group compared to the AD group. Linear regression modeling revealed a significant negative association between P2ry12 and synaptic tau pathology, as well as CD47 expression in p‐tau‐positive synaptosomes. Interestingly, in primary tauopathy microglia (but not AD microglia), IKFZ1‐regulated genes, including P2ry12, were upregulated according to snRNAseq and around p‐tau‐bearing neurons by IHC.

**Conclusion:**

Our data suggest that in normal aging, synaptic Aβ accumulation leads to a diminishing of synaptic CD47 (‘don’t eat me’) signal, accelerating the clearance of malfunctioning Aβ but not p‐tau positive synapses. Upregulation of P2ry12 and CD206, accompanied by downregulation of Clec7a, may represent an early and potentially protective response to pre‐tangle tau pathology. Later in disease progression, P2ry12 reduction may drive a more reactive, phagocytic phenotype in response to tau pathology, leading to an increase in synaptic CD47 levels and synaptic pathology accumulation.

